# Ceramic thin-film composite membranes with tunable subnanometer pores for molecular sieving

**DOI:** 10.1038/s41467-023-42495-w

**Published:** 2023-11-09

**Authors:** Xuechen Zhou, Rahul Shevate, Dahong Huang, Tianchi Cao, Xin Shen, Shu Hu, Anil U. Mane, Jeffrey W. Elam, Jae-Hong Kim, Menachem Elimelech

**Affiliations:** 1 Department of Chemical and Environmental Engineering, Yale University, New Haven, CT USA; 2 Applied Materials Division, Argonne National Laboratory, Lemont, IL USA

**Keywords:** Porous materials, Chemical engineering

## Abstract

Ceramic membranes are a promising alternative to polymeric membranes for selective separations, given their ability to operate under harsh chemical conditions. However, current fabrication technologies fail to construct ceramic membranes suitable for selective molecular separations. Herein, we demonstrate a molecular-level design of ceramic thin-film composite membranes with tunable subnanometer pores for precise molecular sieving. Through burning off the distributed carbonaceous species of varied dimensions within hybrid aluminum oxide films, we created membranes with tunable molecular sieving. Specifically, the membranes created with methanol showed exceptional selectivity toward monovalent and divalent salts. We attribute this observed selectivity to the dehydration of the large divalent ions within the subnanometer pores. As a comparison, smaller monovalent ions can rapidly permeate with an intact hydration shell. Lastly, the flux of neutral solutes through each fabricated aluminum oxide membrane was measured for the demonstration of tunable separation capability. Overall, our work provides the scientific basis for the design of ceramic membranes with subnanometer pores for molecular sieving using atomic layer deposition.

## Abstract (web summary)

Ceramic membranes are a promising alternative to polymeric membranes for selective separations, given their ability to operate under harsh chemical conditions. Here authors demonstrate a molecular-level design of ceramic membranes with tunable subnanometer pores.

## Introduction

The deployment of clean energy technologies is boosting the global metal demand to an unprecedented extent^1,2^. For instance, the annual lithium demand was projected to be 2.5 million tons in 2030, 8.7 times the demand in 2020^3^. With multiple metal demands approaching their global reserves (e.g., 14 million tons for lithium^4^), there is a heightened interest in augmenting the supply of critical metals by either recycling precious metals from industrial waste streams or harvesting metals from unconventional sources such as seawater^5,6^. Recovering valuable metals (e.g., nickel and copper) from waste streams also helps lower the toxicity of the streams and offsets the cost of waste treatment^7,8^. Given the complexity of waste streams, there is a crucial need to develop highly precise separation processes for metal recovery.

Membrane technology has been widely used for selective separations due to its great separation capability^9,10^. Designing polymeric membranes with functional groups favorably interacting with desired ionic species has been demonstrated as an efficient pathway to selectively transport these ions from the feed streams^5,11^. The attraction between membrane pores and ions compensates the energy penalty for ion dehydration at the pore entrance and thus favors their passage^12^. Subnanometer membrane pores are preferred so that the non-selective bulk-phase ion permeation can be minimized^11^. Despite the versatility of the fabrication strategy and the tunability of the membrane chemistry, one major concern regarding employing polymeric membranes for selective separation is their instability under harsh operating conditions (e.g., high or low pH, presence of oxidants, and high temperatures). For instance, polyamide thin-film composite membranes, the state-of-the-art reverse osmosis membranes, are vulnerable to chlorine attack, substantially compromising their separation performance^13–15^.

To further enhance the reliability and operational feasibility of the membrane separation process, it is imperative to design selective membranes with improved stability. One promising alternative is using ceramic membranes (e.g., aluminum oxide^16^, zirconium oxide^17^, and titanium oxide^18^) for selective separation, given their tolerance for harsh chemical conditions such as extreme solution pH and chlorine cleaning^19,20^. Nevertheless, current fabrication techniques, mainly the sol-gel method^21,22^, rely on the assembly of particulates to create the separating layers and thus can only construct microfiltration or ultrafiltration ceramic membranes^23^ (pore size >2 nm). Therefore, to impart precise molecular sieving for ceramic membranes, especially for ion-ion separation, it is crucial to develop ceramic membranes with subnanometer pores.

A molecular-level design of the active layer is required to construct ceramic membranes with subnanometer pores. One proposed strategy is calcinating the metal-organic hybrid films prepared using the molecular layer deposition (MLD) technique^24–26^. Specifically, in these MLD processes, metal atoms and organic linkers are deposited on the substrate surface, layer by layer, by sequentially exposing the substrates to the metal-organic precursors (e.g., titanium tetrachloride) and the bifunctional co-reactants (mainly ethylene glycol, EG). By burning off the organic linkers within the hybrid films, (sub)nanometer pores were supposed to be generated. Despite the attempted molecular-level design, the fabricated ceramic membranes showed limited capability in differentiating between the transport of different ions^26^. This inconsistency is attributable to the condensation of EG during the MLD deposition, creating large defects after calcination^27^. Hence, to realize the application of this sacrificial template strategy in constructing defect-free ceramic membranes with subnanometer pores, it is imperative to build hybrid films with distributed molecular-scale sacrificial segments. However, this objective can barely be realized with the conventional MLD process as EG is among the most volatile bifunctional co-reactants.

In this work, we use monofunctional-alcohol-modulated atomic layer deposition (ALD) to construct aluminum oxide (AlO_x_) membranes with tunable subnanometer pores. Taking the methanol-modulated ALD deposition as an example, we first demonstrate the proposed deposition strategy by monitoring the generation and incorporation of methoxy groups within the deposited films. With the optimized deposition and calcination conditions, we then fabricate AlO_x_ membranes with continuous subnanometer pores by burning off the incorporated methoxy groups. The selectivity of these membranes toward the transport of monovalent and divalent salts is tested. Lastly, permeation of neutral solutes through the AlO_x_ membranes prepared with the modulation of different alcohols (i.e., methanol, ethanol, and isopropanol) is characterized to highlight the tuning of membrane pore size and their separation capability.

## Results

### Methanol-modulated atomic layer deposition of aluminum oxide

**Fig. 1:**
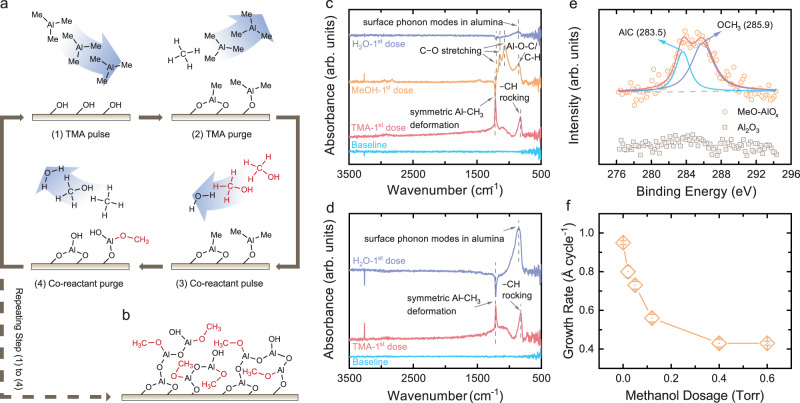
Fabrication of the MeO-AlO_x_ film using the CH_3_OH-modulated ALD process. **a** Schematic diagram describing one ALD cycle of MeO-AlO_x_ deposition, which consists of four steps: (1) TMA pulse; (2) TMA purge; (3) co-reactant pulse (i.e., a mixture of CH_3_OH and H_2_O); and (4) Co-reactant purge. Only a single atomic layer of MeO-AlO_x_ is deposited during one ALD cycle. **b** Schematic diagram illustrating the chemical structure of the deposited MeO-AlO_x_ film, where −OCH_3_ groups (highlighted in red) are incorporated into the Al-O framework during the deposition. **c** Differential FTIR spectra recorded during the CH_3_OH-modulated ALD deposition after one pulse of TMA (red), CH_3_OH (orange), and H_2_O (purple). The original FTIR spectra after each pulse can be found in Supplementary Fig. 1a. **d** Differential FTIR spectra recorded during the standard Al_2_O_3_ ALD deposition after one pulse of TMA (red) and H_2_O (purple). The original FTIR spectra after each pulse can be found in Supplementary Fig. 1b. **e** C 1 *s* XPS spectra of the ALD deposited Al_2_O_3_ film and the CH_3_OH-modulated ALD deposited MeO-AlO_x_ film. Argon sputtering was conducted to remove the adventitious carbon contamination before the XPS test. **f** Dependence of the MeO-AlO_x_ film growth rate on the CH_3_OH dosage during the ALD deposition. For each CH_3_OH dosage, the TMA and H_2_O dosages were fixed at 0.25 and 0.06 Torr, respectively. The growth rate was characterized by measuring the pore narrowing rate when conducting the ALD deposition on top of AAO substrates (Supplementary Figs. 4 and 5). Error bars represent standard deviations from the linear fitting.

A methanol (CH_3_OH)-modulated ALD process was used for precise control of the pore structure within the designed ceramic membranes (Fig. 1). In this ALD process, a methoxy group ( − OCH_3_)-decorated aluminum oxide film (MeO-AlO_x_) can be deposited on the substrate surface by sequentially exposing the substrates to three different chemicals: trimethylaluminum (Al(CH_3_)_3_, TMA), H_2_O, and CH_3_OH (Fig. 1a, b). Compared to EG (HOCH_2_CH_2_OH) used in the MLD process in previous studies, both H_2_O and CH_3_OH are more volatile (boiling points of 100, 64.7, and 198 °C for H_2_O, CH_3_OH, and HOCH_2_CH_2_OH, respectively), reducing the condensation of co-reactants during the deposition (at 150 °C) and the formation of defects after the calcination step^27^.

Figure 1a illustrates a typical cycle of the proposed ALD deposition. In the first half-cycle, hydroxyl groups ( − OH) on the substrate surface react with the dosed TMA precursors and are converted to methyl aluminum groups ( − AlCH_3_). Due to the self-limiting nature of the reaction, only a single layer of −AlCH_3_ groups can be deposited at each cycle. After purging the reaction chamber with argon gas, a mixture of CH_3_OH and H_2_O was dosed sequentially without a purge break, with both co-reactants reacting with the −CH_3_ groups in the following second half-cycle. Specifically, −OH groups are regenerated through the reaction between the H_2_O molecules and the −CH_3_ groups, which can act as the active sites for the next cycle of ALD growth. Concomitantly, the remaining −CH_3_ groups are converted to −OCH_3_ groups after reacting with CH_3_OH; these −OCH_3_ groups inhibit TMA binding in the next ALD cycle. The two sequential reactions in each ALD cycle can be described as follows:

1 $${{{{{\rm{AlOH}}}}}} \ast+{{{{{\rm{Al}}}}}}{({{{{{\rm{C}}}}}}{{{{{{\rm{H}}}}}}}_{3})}_{3}\to {{{{{\rm{AlOAl}}}}}}{({{{{{\rm{C}}}}}}{{{{{{\rm{H}}}}}}}_{3})}_{2} \ast+{{{{{\rm{C}}}}}}{{{{{{\rm{H}}}}}}}_{4}$$

2 $${{{{{\rm{AlOAl}}}}}}{({{{{{\rm{C}}}}}}{{{{{{\rm{H}}}}}}}_{3})}_{2} \ast+{{{{{{\rm{H}}}}}}}_{2}{{{{{\rm{O}}}}}}+{{{{{\rm{C}}}}}}{{{{{{\rm{H}}}}}}}_{3}{{{{{\rm{OH}}}}}}\to {{{{{\rm{AlOAl}}}}}}({{{{{\rm{OC}}}}}}{{{{{{\rm{H}}}}}}}_{3}){{{{{\rm{OH}}}}}} \ast+2{{{{{\rm{C}}}}}}{{{{{{\rm{H}}}}}}}_{4}$$

where the asterisks denote surface species.

In situ Fourier transform infrared (FTIR) spectroscopy was carried out to probe the surface reactions during the proposed ALD deposition, and the surface chemistry was examined after each step of precursor dosage. To explicitly demonstrate the surface change after exposure to the mixture of CH_3_OH and H_2_O, FTIR spectra were obtained separately after sequentially exposing the substrate to CH_3_OH and H_2_O (Supplementary Fig. 1). Differential FTIR spectra were obtained by subtracting the spectra from the corresponding ones acquired in the previous step to highlight the changes occurring during each step (Fig. 1c, d). Positive-going peaks suggest the generation of new species, while negative-going features indicate the consumption of surface functional groups.

According to Fig. 1c, two positive peaks with wavenumbers of 821 and 1209 cm^−1^ appear after the dose of TMA. The peak at 821 cm^−1^ is the characteristic of −CH rocking^28^, and the one at 1209 cm^−1^ represents the symmetric Al−CH_3_ deformation^29^. Both peaks support the generation of −AlCH_3_ groups on the substrate surface after reacting with TMA. Following the dose of CH_3_OH, a negative peak at 1209 cm^−1^ was observed, indicating the consumption of surface −AlCH_3_ groups. Concomitantly, positive peaks with wavenumbers of 1205, 1145, and 1068 cm^−1^ appear, which can be assigned to C–O stretching^30^. The appearance of C–O groups is consistent with the anchoring of −OCH_3_ groups (Fig. 1a). The positive peak appearing at 848 cm^−1^ can be assigned to Al–O–C, affirming the formation of Al–OCH_3_ species. Finally, after the H_2_O dosage, the negative features at 1205, 1145, and 1068 cm^−1^ indicate the partial consumption of −OCH_3_ groups whereas the broad positive peak between 768 and 1077 cm^−1^ represents the surface phonon modes in alumina^30^. The observed spectrum change suggests that the anchored −OCH_3_ groups are vulnerable to H_2_O attack and can be substituted with −OH groups:

3 $${{{{{\rm{AlOC}}}}}}{{{{{{\rm{H}}}}}}}_{3} \ast+{{{{{{\rm{H}}}}}}}_{2}{{{{{\rm{O}}}}}}\to {{{{{\rm{AlOH}}}}}} \ast+{{{{{\rm{C}}}}}}{{{{{{\rm{H}}}}}}}_{3}{{{{{\rm{OH}}}}}}$$

As a control, when we only provided H_2_O after the TMA exposure (i.e., a conventional ALD deposition of Al_2_O_3_; Supplementary Fig. 2), a significantly higher positive peak representing the surface phonon modes in alumina was observed between 768 and 1077 cm^−1^ (Fig. 1d), consistent with the fact that adding CH_3_OH as a co-reactant inhibits the growth of the alumina film.

To further confirm the incorporation of −OCH_3_ groups within the deposited MeO-AlO_x_ film despite the substitution with −OH groups when using the mixture of H_2_O and CH_3_OH as the co-reactant (Eq. 3), we characterized the chemical composition of the deposited film using X-ray photoelectron spectroscopy (XPS). In situ argon ion sputtering was applied before the XPS measurement to clean adventitious carbon so that the carbon content within the deposited films can be explicitly detected. According to Fig. 1e, carbon species were found within the deposited MeO-AlO_x_ film, whereas no significant peak was observed within the C 1*s* XPS spectrum of the Al_2_O_3_ film deposited in the absence of CH_3_OH. Additional deconvolution of the C 1 *s* peak reveals that the detected carbon species within the MeO-AlO_x_ film has two components. Specifically, the peak at 285.9 eV is consistent with previously reported oxygen-bound carbon within −OCH_3_ groups^31^, and the other peak with binding energy of 283.5 eV can be ascribed to the metal carbide (Al–C here) which was generated during the argon sputtering process^32^.

To demonstrate the tuning of −OCH_3_ group density within the deposited films, the film growth rates at various CH_3_OH dosages were characterized (Supplementary Figs. 4 and 5). As shown in Fig. 1f, when H_2_O was used as the only co-reactant (i.e., 0 Torr of CH_3_OH dosage), the growth rate of the film was 0.95 Å cycle^−1^, in agreement with previously reported Al_2_O_3_ ALD film growth rate at 150 °C^33,34^. As the CH_3_OH dosage increases during the ALD deposition, the film growth rate decreases continuously, suggesting that more −CH_3_ groups are converted to −OCH_3_ groups (terminators) rather than −OH groups (active sites) during the co-reactant exposure. When the CH_3_OH dosage is higher than 0.40 Torr, the film growth rate is almost constant ( ~ 0.40 Å cycle^−1^), likely due to the near saturation of the substrate surface with −OCH_3_ groups^35^.

### Fabrication of aluminum oxide thin-film composite membranes

**Fig. 2:**
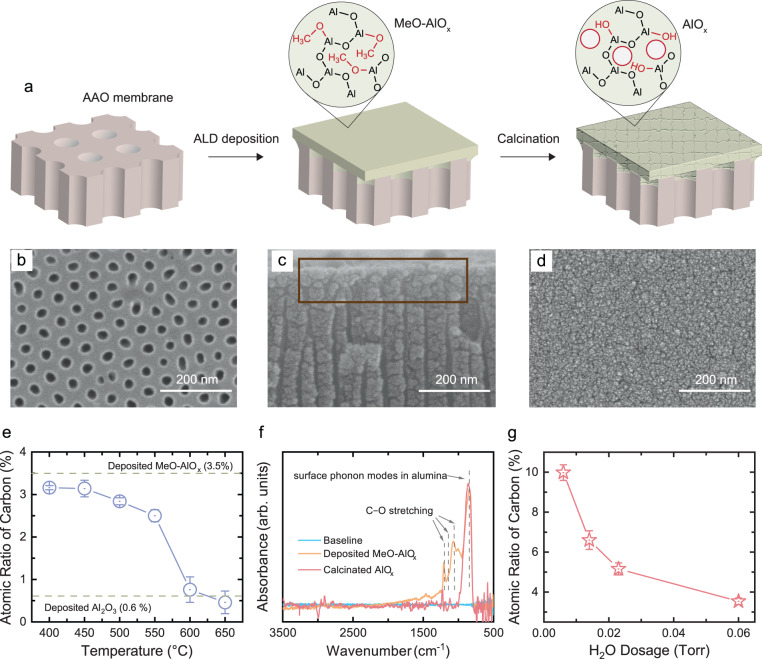
Construction of the AlO_x_ TFC membranes with subnanometer pores. **a** Schematic diagram showing the fabrication of the AlO_x_ TFC membranes. Briefly, a continuous MeO-AlO_x_ film was deposited on top of the AAO substrate using ALD. This composite membrane was then calcinated to burn off the −OCH_3_ groups to create continuous pathways for water and solute permeation. **b** SEM image of the top surface of the pristine AAO substrate. **c** SEM image of a cross-section of the MeO-AlO_x_ TFC membrane. A continuous integral layer (highlighted in the brown box) was observed near the top of the AAO substrate. **d** SEM image of the top surface of the AlO_x_ TFC membrane. Scale bars are 200 nm. **e** Dependence of the carbon atomic ratio within the ALD deposited MeO-AlO_x_ film on the calcination temperature. All the carbon species are burnt off at calcination temperatures higher than 600 °C. Error bars represent standard deviations of 5 measurements. **f** FTIR spectra of the ALD deposited MeO-AlO_x_ film and the AlO_x_ film after being calcinated at 600 °C. **g** The impact of H_2_O dosage during the ALD deposition on the atomic ratio of carbon within the deposited MeO-AlO_x_ ALD layers. For each H_2_O dosage, the TMA and CH_3_OH dosages were fixed at 0.25 and 0.40 Torr, respectively. Error bars represent standard deviations of 5 measurements.

As illustrated in Fig. 2a, an anodic aluminum oxide (AAO) membrane was used as the substrate for membrane fabrication, and a continuous MeO–AlO_x_ film was constructed on top of the substrate using the proposed ALD process. The ALD coating first narrows the pores of the AAO substrate and then forms a continuous layer^36^. The minimum number of ALD cycles required to completely block the AAO pores was calculated based on the measured pore radius (~15.4 ± 0.7 nm, Supplementary Fig. 5) and the film growth rate (depending on the precursor dosage, Fig. 1f). Scanning electron microscope (SEM) was used to verify the construction of the continuous MeO-AlO_x_ layer; compared to the pristine AAO substrate (Fig. 2b), no microscopically visible pore structure was observed on top after the ALD coating (Supplementary Fig. 6a). A cross-sectional SEM image shows that the ALD coating only spans the top ~100 nm of the AAO substrate (Fig. 2c), suggesting a thin-film composite (TFC) structure of the constructed membranes. This observation agrees well with the small TMA dose time (0.07 s) we employed for deposition, which is significantly lower than that required for the AAO pore saturation (calculated saturation dose time: 14.7 s; Supplementary Note 1). In addition, the progressive decrease in pore diameter with increasing ALD cycles adds significant resistance to TMA diffusion inside the AAO pores, resulting in the formation of the TFC structure on AAO substrates^37^.

The fabricated composite membrane was then calcinated to burn off the −OCH_3_ groups to create pathways to both water and solute transport (Fig. 2a, denoted as AlO_x_ membranes). No visible destruction of the ALD layer was observed after being calcinated for 5 h under an air atmosphere (Fig. 2d), and the membranes became hydrophilic (Supplementary Fig. 6b). The calcination temperature was optimized to ensure the complete removal of the −OCH_3_ groups. Briefly, the deposited MeO-AlO_x_ films were calcinated at different temperatures and their chemical composition after the calcination, especially the atomic carbon ratio, was characterized using the XPS technique. As shown in Fig. 2e, carbon was found to make up 3.5% of the deposited film, and this ratio was maintained nearly constant when the film was calcinated at a temperature lower than 450 °C. Raising the calcination temperature above 450 °C resulted in a continuous decline of the atomic ratio of carbon within the film. When the calcination temperature was higher than 600 °C, no residual carbon was observed (Supplementary Fig. 7), indicating that most of the −OCH_3_ groups within the deposited ALD layer were burned off. The removal of the −OCH_3_ groups after the calcination was further examined using FTIR. According to the FTIR spectra in Fig. 2f, peaks assigned to C−O stretching (wavenumber of 1205, 1145, and 1068 cm^−1^) disappeared after the calcination of the deposited MeO-AlO_x_ film at 600 °C. Hence, the calcination temperature was determined to be 600 °C for membrane fabrication.

The −OCH_3_ group density is mainly controlled by the CH_3_OH and H_2_O dosage. It is noteworthy that even when CH_3_OH was overdosed (pulse pressure of 0.40 Torr compared to 0.06 Torr of H_2_O), the atomic ratio of carbon within the deposited film was only 3.5% (characterized by XPS; Fig. 2g). Considering that each carbon atom is surrounded by ~19.8 oxygen atoms and ~9.5 aluminum atoms within this film (Supplementary Fig. 8), continuous transport pathways can hardly be formed even if all the −OCH_3_ groups are burned off. This low density of −OCH_3_ groups can be attributed to either the greater reactivity of H_2_O compared to the CH_3_OH molecules, or the replacement of −OCH_3_ groups by −OH groups according to Eq. 3. Therefore, to further increase the −OCH_3_ group density, the H_2_O dosage was adjusted. As summarized in Fig. 2g, when the H_2_O pulse pressure was decreased from 0.06 (i.e., the typical H_2_O pulse for the Al_2_O_3_ ALD deposition) to 0.007 Torr, the atomic ratio of carbon within the deposited films increased from 3.5% to 10.0%. Specifically, for the MeO-AlO_x_ film deposited with 0.007 Torr of H_2_O dosage, each carbon atom is surrounded by ~6.1 oxygen atoms and ~ 2.9 aluminum atoms (Supplementary Fig. 8), which is more promising for the creation of continuous transport pathways within the membrane compared to the film deposited with 0.06 Torr of H_2_O dosage.

### Transport and separation properties of the aluminum oxide membranes

**Fig. 3:**
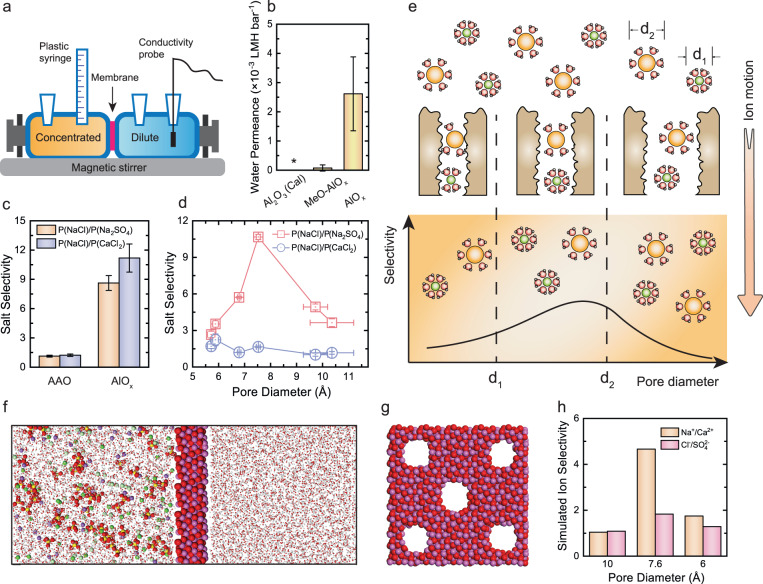
Selective ion transport through the AlO_x_ ALD membranes with subnanometer pores. **a** Schematic diagram illustrating the customized diffusion cell used for the transport tests. **b** Water permeability coefficient of the calcinated AlO_x_ membranes (right), the deposited MeO-AlO_x_ films (middle), and the calcinated Al_2_O_3_ membranes deposited by the conventional Al_2_O_3_ ALD deposition (left). Error bars represent standard deviations from measurements with three different membranes. **c** Selectivity between NaCl and Na_2_SO_4_, and NaCl and CaCl_2_, determined from salt permeation through the AAO substrates and the composite AlO_x_ membranes. 0.1 M single salt and DI water were used as the concentrated and dilute solutions, respectively. Error bars represent standard deviations from measurements with three membranes. **d** Dependence of the selectivity between monovalent salts (i.e., NaCl) and divalent salts (i.e., Na_2_SO_4_ and CaCl_2_) on the size of subnanometer pores. Polyamide TFC membranes with varied pore diameters were mounted into the diffusion cell. For these experiments, 0.1 M single salt solutions were used as the concentrated solutions, whereas 0.2 and 0.3 M sucrose solutions were used as the dilute solutions to balance the osmotic pressure during the measurements with monovalent and divalent salts, respectively. Error bars represent standard deviations from measurements with three membranes. **e** Schematic diagram illustrating the selective transport of hydrated ions through pores of varied sizes. **f** Schematic of the simulation box consisting of a salt solution with 1.0 M of Na^+^, Ca^2+^, Cl^−^, and SO_4_^2−^ ions (left), an AlO_x_ ALD membrane (middle), and pure H_2_O (right). **g** Cross-sectional view of an AlO_x_ ALD membrane with 7.6 Å pores. Five pores were drilled such that significant ion flux can be obtained in a concentration-gradient-driven process. **h** Simulated monovalent and divalent selectivity (Na^+^/Ca^2+^ and Cl^−^/SO_4_^2−^) through AlO_x_ membranes with pore diameters of 6, 7.6, and 10 Å.

The transport properties of the fabricated AlO_x_ membranes were evaluated using a customized two-chamber diffusion cell (Fig. 3a). Specifically, water permeation was characterized to rationalize our design and verify the formation of continuous voids within the calcinated AlO_x_ films. As shown in Fig. 3b, a notably higher water permeance was observed for the calcinated AlO_x_ membranes (2.6 × 10^−3^ LMH bar^−1^) compared to the ALD deposited MeO-AlO_x_ films (both deposited with 0.007 Torr of H_2_O dosage), suggesting that transport pathways can be created via burning off the decorated −OCH_3_ groups. No apparent water flux was observed for the calcinated AlO_x_ membranes created with 0.06 Torr of H_2_O dosage. Therefore, the pulse pressure during membrane construction was set at 0.4 and 0.007 Torr for CH_3_OH and H_2_O dosage, respectively. Densification caused by the phase transition of thin films at high temperatures, i.e., from amorphous to crystalline, has been reported to contribute to the pore formation as well^38^. However, this is not the main mechanism here as no water transport was observed with the Al_2_O_3_ ALD films calcinated at the same temperature (600 °C; Fig. 3b).

The selectivity of the fabricated AlO_x_ membranes for the transport of monovalent and divalent salts was then evaluated using the same concentration-gradient-driven process. Single salt solutions (NaCl, CaCl_2_, and Na_2_SO_4_) were placed on the concentrated side and the salt permeation rate was calculated by monitoring the increase in electric conductivity on the dilute side. As summarized in Fig. 3c, a significantly higher permeation rate was observed for NaCl over the permeation rate of Na_2_SO_4_ and CaCl_2_ (i.e., salt selectivities of 8.6 and 11.2, respectively). As a comparison, the pristine AAO substrates showed limited capability in differentiating the transport of these solutes, and a ratio of ~1.2:1 was observed between the NaCl flux and the flux of the divalent salts (Fig. 3c). The slightly faster NaCl permeation through the nanopores of the AAO substrates is attributable to the higher diffusion coefficients of monovalent ions (1.334 × 10^–9^ and 2.032 × 10^–9^ m^2^ s^–1^ for Na^+^ and Cl^−^ ions, respectively^39^) compared to divalent ions (7.9 × 10^–10^ and 1.065 × 10^–9^ for Ca^2+^ and SO_4_^2−^ ions, respectively^39^). Thus, the observed fast NaCl permeation with the constructed AlO_x_ TFC membranes is contributed by the AlO_x_ active layer.

Electrostatic interactions have been proposed to govern the selective transport of ions with different charges through nanopores^40^. For example, divalent cations (e.g., Ca^2+^) were observed to permeate faster through negatively charged pores compared to the monovalent cations (e.g., Na^+^), which was explained by the higher electrostatic attraction between the divalent cations and the pores^40^. The permeation of divalent cations became slower when pores were positively charged^41^. This electrostatic effect can explain our observed faster Na_2_SO_4_ permeation through the AlO_x_ membranes compared to CaCl_2_ (i.e., lower NaCl/Na_2_SO_4_ selectivity compared to NaCl/CaCl_2_ selectivity in Fig. 3c), as AlO_x_ is positively charged at the experimental pH (5.7)^42,43^. Nevertheless, an 8.6-time-higher NaCl flux was observed compared to Na_2_SO_4_ flux, suggesting that electrostatic interaction is not the dominant mechanism contributing to the observed selective ion transport. In other words, the ultrahigh monovalent/divalent selectivity obtained with the AlO_x_ membranes should be attributed to the steric confinement within the active layer.

To further demonstrate the role of pore size in differentiating the transport of monovalent and divalent salts, the permeation rate of NaCl, CaCl_2_, and Na_2_SO_4_ through polyamide (PA) TFC membranes with different pore diameters was measured. It is worth noting that although the observed selectivity with the fabricated AlO_x_ membranes cannot be fully interpreted by the experiments with PA TFC membranes, the PA TFC membranes, ranging from reverse osmosis (RO) to ultrafiltration (UF), provide a unique platform to study the size-dependent transport phenomenon at the subnanometer scale. Notably, the highest selectivity between Cl^−^ and SO_4_^2−^ ions was obtained with the 7.5 Å pores (i.e., NaCl/Na_2_SO_4_ selectivity of 10.7, Fig. 3d); this selectivity declined for membranes with smaller pores and membranes with larger pores. This observed dependence of ion-ion selectivity on the pore size is different from our expectation, where a monotonic increase of the selectivity should be obtained with enhanced confinement (i.e., with smaller pore size) due to the higher extent of ion dehydration. Considering that SO_4_^2−^ and Cl^−^ ions have respectively hydrated diameters of 7.6 and 6.6 Å^44,45^, this observed selectivity trend suggests that the state of hydration/dehydration, rather than the extent of dehydration (i.e., the number of surrounded H_2_O molecules lost), plays the predominant role in determining the selective ion transport (Fig. 3e)^46^.

Specifically, in the case of ion permeation through pores larger than SO_4_^2−^ ions, both SO_4_^2−^ and Cl^−^ ions permeate quickly with an intact hydration shell, resulting in a limited ion-ion selectivity^47^. For ions permeating through pores similar to the size of the SO_4_^2−^ ions, SO_4_^2−^ ions have to adjust their hydration shell and thus need to overcome a high resistance during both pore entry and diffusion inside the pore. However, the smaller Cl^−^ ions can still permeate through the pore rapidly without dehydration, leading to a high Cl^−^/SO_4_^2−^ selectivity^47^. When the pores are smaller than the size of Cl^−^ ions, both SO_4_^2−^ and Cl^−^ ions must undergo significant dehydration and the selectivity between their transport is limited^47^. This pore-size-dependent dehydration-based selective ion transport only holds when there is no specific interaction between the pore wall and ions. Notably, despite Ca^2+^ ions (diameter of 8.2 Å^48^) being more prone to dehydration compared to Na^+^ ions (diameter of 7.0 Å^48^), limited selectivity between NaCl and CaCl_2_ was observed with the PA TFC membranes as the strong interaction of Ca^2+^ with −COO^−^ functional groups in the PA membrane can compensate the dehydration energy of Ca^2+^ ions. This size-dependent ion selectivity also suggests that the average pore diameter of the fabricated AlO_x_ membranes was ~7.6 Å, given that these membranes showed an exceptional capability in differentiating the transport of SO_4_^2−^ and Cl^−^ ions and the transport of Ca^2+^ and Na^+^ ions.

Molecular dynamics (MD) simulations were then performed to verify the dominant role of ion hydration/dehydration in differentiating the transport of monovalent and divalent ions. Salt solutions containing Na^+^, Ca^2+^, Cl^−^, and SO_4_^2−^ ions and pure H_2_O were placed on two sides of the AlO_x_ membrane (Fig. 3f). Five pores with identical diameters were drilled to facilitate the ion flux in a concentration-gradient-driven process (Fig. 3g). The selectivity between monovalent and divalent ions was calculated for AlO_x_ pores with diameters of 10, 7.6, and 6 Å to study the size-dependent ion selectivity.

When the pore diameter was 10 Å, all four ions (Na^+^, Ca^2+^, Cl^−^, and SO_4_^2−^) retained a similar hydration shell within the pores compared to their respective hydration shell in the bulk solution (Supplementary Fig. 10). Consequently, the selectivity between monovalent and divalent ions was close to 1 (Na^+^/Ca^2+^ = 1.0 and Cl^−^/ SO_4_^2−^ = 1.1; Fig. 3h). When the pore diameter was narrowed to 7.6 Å, divalent ions partially lost surrounding H_2_O molecules during their passage through the membrane pores while monovalent ions still maintained their original hydration shell (Supplementary Fig. 10), leading to an increased monovalent/divalent selectivity (Na^+^/Ca^2+^ = 4.7 and Cl^−^/ SO_4_^2− ^= 1.9; Fig. 3h). Further narrowing the pore diameter to 6 Å enforced both the monovalent and divalent ions to adjust their hydration shell (Supplementary Fig. 10), resulting in a lower selectivity between them (Na^+^/Ca^2+^ = 1.8 and Cl^−^/ SO_4_^2− ^= 1.3; Fig. 3h).

### Tuning the pore size of the aluminum oxide membranes for molecular sieving

**Fig. 4:**
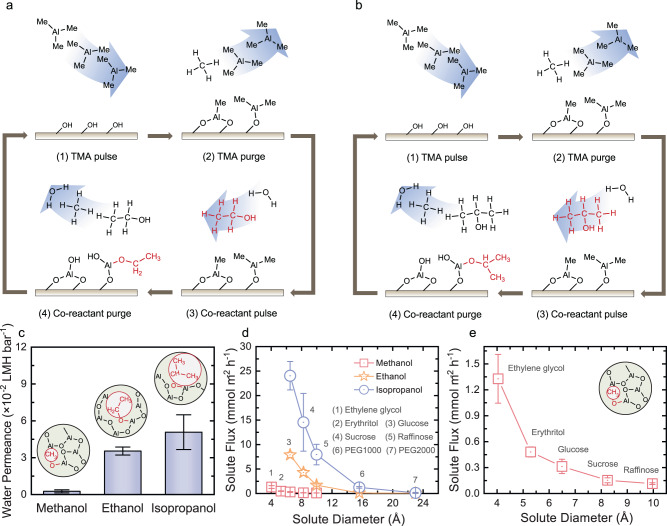
Tuning the subnanometer pores of the AlO_x_ membranes for molecular sieving. Schematic diagrams of **a** ethanol-modulated and **b** isopropanol-modulated ALD deposition. Both processes consist of four steps: (1) TMA pulse; (2) TMA purge; (3) co-reactant pulse (a mixture of H_2_O and ethanol for ethanol-modulated process, and a mixture of H_2_O and isopropanol for isopropanol-modulated process); and (4) co-reactant purge. **c** Water permeability coefficient of the AlO_x_ membranes fabricated with methanol, ethanol, and isopropanol as co-reactants. In each measurement, 1 M NaCl was utilized as the concentrated solution and DI water was used on the dilute side. Error bars represent standard deviations from measurements with three membranes. **d** Permeation rate of neutral solutes through the AlO_x_ membranes with tunable subnanometer pores. For each test, 0.1 M single solute solutions were placed on the concentrated side and the permeation rate was calculated by measuring the TOC increase on the permeate side. Error bars represent standard deviations from measurements with at least two membranes. **e** Amplification of the solute flux through the AlO_x_ membranes fabricated with methanol in **d**.

Pore structure within the constructed AlO_x_ membranes was adjusted by varying the co-reactants during the monofunctional-alcohol-modulated ALD deposition. Specifically, ethanol (CH_3_CH_2_OH; 0.2 Torr) and isopropanol ((CH_3_)_2_CHOH; 0.2 Torr) were employed to incorporate ethoxy ( − OCH_2_CH_3_) and isopropoxy ( − OCH(CH_3_)_2_) groups into the deposited films, respectively (Fig. 4a, b). These materials were deposited on top of the AAO substrates to construct pinhole-free films. We then calcinated the hybrid films to burn off carbonaceous species of different dimensions to create active layers with varied pore sizes. According to Fig. 4c, when the co-reactants were switched from CH_3_OH to CH_3_CH_2_OH and (CH_3_)_2_CHOH, the water permeability of the fabricated membranes increased from 2.6 × 10^−3^ to 3.5 × 10^−2^ and 5.1 × 10^−2^ LMH bar^−1^, respectively. The enhanced water permeability suggests that larger pores were created when we burned off the −OCH_2_CH_3_ and −OCH(CH_3_)_2_ groups within the deposited films.

To further estimate the membrane pore size variation when using different alcohols as co-reactants during the deposition and demonstrate the tunable capabilities of the AlO_x_ membranes in separating solute transport, the permeation rate of neutral solutes of various sizes through each fabricated membrane was measured. Neutral solutes were used here to exclude the impact of other mechanisms (e.g., Donnan and dielectric^49^) on the solute transport such that the impact of size can be explicitly demonstrated. A hyperbolic dependence of solute flux on their radius was observed with the membranes created with CH_3_OH, CH_3_CH_2_OH, and (CH_3_)_2_CHOH, consistent with the size-exclusion-dominating selective solute transport (Fig. 4d).

For the AlO_x_ membranes created with CH_3_OH (Fig. 4d, e), limited transport was observed when the solute diameter is larger than 8.1 Å (sucrose of 8.2 Å and raffinose of 10.0 Å). When the solute diameter is smaller than 6.6 Å (glucose of 6.5 Å, erythritol of 5.3 Å, and ethylene glycol of 4.0 Å), these solutes can transport through the membrane pores, and their permeation rate increased with the decreasing solute diameter. The observed solute flux suggests that the average pore diameter of the membranes fabricated with CH_3_OH is between 6.6 and 8.1 Å, consistent with the average pore diameter estimated based on the selective transport between monovalent and divalent ions ( ~ 7.6 Å).

The flux of neutral solutes through the AlO_x_ membranes increased when CH_3_CH_2_OH was utilized as the co-reactant compared to CH_3_OH. For instance, the flux of glucose increased from 0.31 to 7.9 mmol m^−2^ h^−1^. More significantly, sucrose and raffinose can permeate through the AlO_x_ membranes fabricated with CH_3_CH_2_OH with respective fluxes of 4.3 and 1.7 mmol m^−2^ h^−1^, confirming that larger pores were created by burning out the −OCH_2_CH_3_ groups. We note that negligible permeation of polyethylene glycol 1000 (PEG 1000) was observed with the membranes created with CH_3_CH_2_OH, indicating that the average pore diameter of these membranes is between 10.1 and 15.5 Å.

When (CH_3_)_2_CHOH was used as the co-reactant, the solute flux through the constructed AlO_x_ membranes further increased. For example, the flux of raffinose increased from 1.7 to 8.0 mmol m^−2^ h^−1^ when the co-reactants were switched from CH_3_CH_2_OH to (CH_3_)_2_CHOH. Furthermore, PEG 1000 was observed to permeate through the membranes fabricated with (CH_3_)_2_CHOH with a flux of 1.3 mmol m^−2^ h^−1^. This neutral solute transport measurement suggests that the average pore size of the AlO_x_ membranes constructed with (CH_3_)_2_CHOH is between 15.7 and 23.0 Å. Overall, the observed dependence of solute flux on their size shows the feasibility of using this proposed ALD strategy to construct membranes with tunable pore sizes for molecular sieving.

## Discussion

We demonstrated the construction of AlO_x_ membranes with tunable subnanometer pores by burning off the carbonaceous functional groups within the hybrid films prepared using a monofunctional-alcohol-modulated ALD process. This molecular-level design of the pore structure enables the application of ceramic membranes in precise ion separations with subnanometer confinement. Specifically, the AlO_x_ membranes fabricated with CH_3_OH can selectively transport NaCl 8.6 times faster compared to Na_2_SO_4_, matching the highest selectivity obtained with state-of-the-art polyamide membranes (10.7:1 with NF270). The observed slower passage of Na_2_SO_4_ is attributable to the enforced dehydration of SO_4_^2−^ ions within the membrane pores. As a comparison, Cl^−^ ions can permeate through the pores quickly with an intact hydration shell. Further adjusting the co-reactants during the ALD deposition creates AlO_x_ membranes with varied pore sizes, enhancing the versatility of ceramic membranes for precise molecular sieving. Future work could focus on using other metal precursors to construct ceramic membranes with varied surface chemistry. Such membranes will enable the systematic investigation of the role of ion-membrane chemical interactions in selective ion transport.

## Methods

### Materials and chemicals

AAO templates (pore diameter between 20 and 30 nm according to the manufacturer, 60 µm in thickness) were purchased from Hefei Pu-Yuan Nano Technology Co., Ltd. (China). Commercial SW30 XLE, XLE, NF90, NF270, UA60, and NDX membranes were purchased from Sterlitech Corporation (Auburn, WA). TMA (99.999%), CH_3_OH ( ≥ 99.9%), CH_3_CH_2_OH ( ≥ 99.9%), (CH_3_)_2_CHOH ( ≥ 99.9%), NaCl ( ≥ 99.5%), Na_2_SO_4_ (≥99.0%), CaCl_2_ (≥99.0%), EG ( ≥ 99.0%), erythritol ( ≥ 99.0%), glucose ( ≥ 99.0%), sucrose ( ≥ 99.5%), raffinose ( ≥ 98.0%), PEG 1000, and PEG 2000 were purchased from Sigma-Aldrich (St. Louis, MO). Deionized water ( > 18.2 MΩ cm) was obtained from a Milli-Q ultrapure water purification system (Integral 10, Millipore, Billerica, MA).

### Aluminum oxide thin-film composite membrane fabrication

AAO substrates were loaded as received into the ALD reaction chamber (Fiji G2, Veeco-CNT) and preheated at 150 °C for 30 min. The ALD deposition of carbon-decorated aluminum oxide materials was conducted with the sequential pulse of TMA and the co-reactant consisting of a mixture of monofunctional alcohol (CH_3_OH, CH_3_CH_2_OH, or (CH_3_)_2_CHOH) and water. Argon (99.999997%) gas was supplied to carry the precursors into the reaction chamber and to purge the chamber to remove the unreacted precursors after each step of the reaction. The TMA pulse pressure was fixed at 0.25 Torr, and the dosage of alcohol and water was adjusted. All the precursors were maintained at room temperature during deposition. This cycle was repeated to construct a continuous film (17 nm in thickness) on top of the AAO substrates. Porous AlO_x_ layers were then generated by calcinating the deposited hybrid layers in a muffle furnace (Thermolyne, Thermo Fisher Scientific, Waltham, MA) for 5 h under an air atmosphere. The ramp rate was maintained at 1 °C min^−1^ and the calcinating temperature was optimized between 400 and 650 °C to burn off the carbonaceous species within the deposited film.

### Material characterization

In situ FTIR measurement was performed in transmission mode on a Nicolet 6700 FTIR spectrometer (Thermo Scientific) interfaced with a custom-built viscous flow ALD reactor. ZrO_2_ powders ( < 100 nm diameter) pressed into a stainless-steel mesh (Fotofab, Chicago, IL) were used as the substrates for FTIR analysis. The mesh was mounted into the FTIR stage and equilibrated inside the ALD reactor before the FTIR measurement. The growth rate of the ALD material and the morphology of the constructed AlO_x_ composite membrane were characterized via scanning electron microscopy (SEM; SU8230, Hitachi, Japan). For growth rate characterization, SEM images of the AAO substrates undergoing different cycles of ALD modification were captured (500k magnification) and the pore radius of each sample was analyzed using ImageJ (averaged from >20 measurements). The film growth rate was calculated by monitoring the dependence of the AAO pore radius on the number of ALD cycles conducted. The elemental composition of the ALD deposited film and the film undergoing calcination was characterized by XPS spectroscopy (VersaProbe II, Physical Electronics, Chanhassen, MN) using Al Kα radiation (hν = 1486.6 eV). To eliminate the impact of the adventitious carbon on the analysis, Ar sputtering was conducted with a power of 1 kV before the XPS measurement. The binding energy of each element was calibrated by setting the binding energy of the O 1 *s* peak to 530.0 eV.

### Membrane transport test

The transport properties of the fabricated AlO_x_ membranes were evaluated using a homemade diffusion cell (Fig. 3a). Each chamber of the cell has a volume of ~60 mL. A membrane coupon with an effective area of 0.64 cm^2^ was assembled between these two chambers with the AlO_x_ active layer facing the concentrated side. To test the water permeation of the fabricated composite membranes, 1 M NaCl and DI water were used as the concentrated and dilute solutions, respectively. Water flux was measured by monitoring the volume increase of the concentrated chamber using a graduated plastic syringe. The ability of the fabricated AlO_x_ membranes to differentiate the transport of different solutes was evaluated by using 0.1 M solutes as the concentrated solutions (i.e., NaCl, CaCl_2_, Na_2_SO_4_, erythritol, xylose, glucose, sucrose, raffinose, PEG 1000, and PEG 2000). Salt concentration in the permeate side was monitored using the conductivity probe, and the concentration of neutral solutes was measured by a total organic carbon analyzer (TOC-VCSH, Shimadzu, Japan). All measurements were conducted at 22 °C with vigorous mixing in both chambers.

### Molecular dynamics simulations

The simulation box consisted of three main components: an amorphous Al_2_O_3_ membrane with subnanometer pores, a salt solution containing H_2_O, Na^+^, Ca^2+^, Cl^−^, and SO_4_^2−^ ions, and pure H_2_O. To create the amorphous Al_2_O_3_, a sample of crystalline Al_2_O_3_ (downloaded from the Materials Project) was heated at 3000 K for 100 ps using Materials Studio in Forcite Module with COMPASSII as the force field. The amorphous Al_2_O_3_ was then generated using the *supercell* function. The salt solution and pure H_2_O were constructed using Amorphous Cell and combined with the amorphous Al_2_O_3_ using the *build* function.

The molecular structure of the simulation box was optimized using the Forcite Module with COMPASSII as the force field. During the geometric optimization, the convergence threshold for maximum energy change, maximum force, and maximum displacement were set to 0.001 kcal mol^−1^, 0.5 kcal mol^−1^ Å^−1^, and 0.015 Å, respectively. To release the internal stress in the system, an MD simulation was performed under the NPT ensemble for 100 ps at 0.0001 GPa and 298 K until the density was stable over time. Further optimization of the system involved an MD simulation under the NVT ensemble for 100 ps. The simulation of ion passage involved the calculation of electrostatic interaction using Ewald and van der Waals force using Atom base. Precise Nose-Hoover temperature control mode and Berendsen pressure control mode were used in the simulation, and the concentration of ions permeating the membranes was calculated at the end of the MD simulation. All the simulations were conducted at the temperature of 298 K with a step size of 0.5 fs.

### Supplementary information

Supplementary Information

Peer Review File

## Data Availability

The data that support the findings of this study are available from the corresponding authors upon request.
