# Supplementary material for: Ceramic thin-film composite membranes with tunable subnanometer pores for molecular sieving
Source: Nat Commun. 2023 Nov 9;14:7255. doi: 10.1038/s41467-023-42495-w (PMC10636005; doi:10.1038/s41467-023-42495-w)
Supplement: Supplementary file 1 — Supplementary Information [file 41467_2023_42495_MOESM1_ESM.pdf]

Supplementary Information

Ceramic thin-film composite membranes with tunable subnanometer pores for molecular sieving

Xuechen Zhou et al.

Supplementary Note 1.

The minimum exposure time (t) of metal-precursor vapors required for AAO substrate pore saturation can be estimated by Supplementary Eq. 1[1]:

$$t = 2.3 \times 10^{-7} P^{-1} m^{1/2} \Gamma (L/d)^2 \quad (1)$$

where P is partial pressure of the metal precursor ($P = 0.25$ Torr); m is the molecular weight of metal precursor ($m = 72$ g mole^{-1}); Γ density of ALD reactive sites in 10^{15} cm^{-2} ($\Gamma = 0.50$); L is the thickness (L = 60 µm) and d is the pore diameter ($d = 30.8$ nm) of the AAO substrate.

Supplementary Note 2.

The average pore radius of the polyamide thin-film composite membranes used in this study (SW30, XLE, NF90, NF270, NDX, and UA60) was estimated based on the pore hindrance transport model described in our previous study.[2, 3] In this model, membranes are assumed to have a bundle of cylindrical capillary pores with the same radius. The retention of membranes to spherical particles (R_r) can be described as

$$R_\mathrm{r} = 1 - \frac{c_\mathrm{L}}{c_0} = 1 - \frac{\Phi K_\mathrm{c}}{1 - \exp(-\mathrm{Pe})(1 - \Phi K_\mathrm{c})} = 1 - \frac{\Phi K_\mathrm{c}}{1 - \exp\left(-\frac{\mathrm{Pe}}{J_\mathrm{V}} J_\mathrm{V}\right)(1 - \Phi K_\mathrm{c})} \quad (2)$$

where c_L and c_0 are the particle concentrations just outside the pore entrance and exit; Φ is the distribution coefficient for hard-sphere particles when only steric interactions are considered:

$$\Phi = (1 - \lambda)^2 \quad (3)$$

where λ is the ratio of the particle radius to pore radius, $\lambda = r_\mathrm{s}/r_\mathrm{p}$; Pe is the membrane Peclect number defined as:

$$\mathrm{Pe} = \frac{K_\mathrm{c} J_\mathrm{V} L}{K_\mathrm{d} \epsilon D_\infty} \quad (4)$$

where K_c is the hydrodynamic hindrance coefficient for convection; K_d is the hydrodynamic hindrance coefficient for diffusion; J_V is the membrane volumetric permeate flux; D_∞ is the Stokes-Einstein diffusion coefficient; L is the entire pore length or membrane active layer thickness; ϵ is the membrane porosity.

The K_c and K_d can also be related to the ratio of particle radius to pore radius (λ) according to Bungay and Brenner's work[4]:

$$K_\mathrm{c} = \frac{[2 - (1-\lambda)^2]\{\frac{9}{4}\pi^2\sqrt{2}(1-\lambda)^{-\frac{5}{2}}[1 + \sum_{n=1}^{2} b_n (1-\lambda)^n] + \sum_{n=0}^{4} b_{n+3}\lambda^n\}}{2\{\frac{9}{4}\pi^2\sqrt{2}(1-\lambda)^{-\frac{5}{2}}[1 + \sum_{n=1}^{2} a_n (1-\lambda)^n] + \sum_{n=0}^{4} a_{n+3}\lambda^n\}} \quad (5)$$

$$K_\mathrm{d} = \frac{6\pi}{\frac{9}{4}\pi^2\sqrt{2}(1-\lambda)^{-\frac{5}{2}}\left[1+\sum_{n=1}^{2} a_n(1-\lambda)^n\right]+\sum_{n=0}^{4} a_{n+3}\lambda^n} \quad (6)$$

The coefficients a_n and b_n, for up to n=7, can also be found in ref 4.

Hence, in this model R_r of spherical particles only depends on J_V, λ, L, and ϵ.

Meanwhile, R_r can also be achieved experimentally. Neutral organic molecules (ethylene glycol, erythritol, and glucose) were employed as solutes and were assumed to be spherical particles. We dissolved the individual organic molecules into the feed solution to obtain a feed concentration of 50 mg/L (as total organic carbon (TOC)). Membrane coupons (area of 20 cm^2) were mounted into a customized crossflow filtration system and were compacted at high pressure (31.7 bar for SW30, XLE, and NF90, and 20.7 bar for NF270, NDX, and UA60) for over 8 hours before the rejection test. Permeate was collected at the pressure of 27.6, 20.7, 13.8, and 6.9 bar for SW30, XLE, and NF90 membranes and at the pressure of 17.2, 13.8, 10.3, and 6.9 bar for NF270, NDX, and UA60 membranes. The crossflow rate was maintained at 21.4 cm/s. After adjusting the pressure, the system was stabilized for 1 hour before sample collection. Membrane permeate flux was recorded at each pressure. The exact solute concentration in the feed solution and permeate was analyzed by the TOC analyzer (TOC-VCSH, Shimadzu, Japan) to get the observed solute rejection R_o.

According to the film theory for concentration polarization, the R_r can be calculated via

$$\ln\frac{1-R_\mathrm{r}}{R_\mathrm{r}} = \ln\frac{1-R_\mathrm{o}}{R_\mathrm{o}} - \frac{J_\mathrm{V}}{k_\mathrm{f}} \quad (7)$$

where k_f is the mass transfer coefficient. In our system ($\mathrm{Re} = 2254$, turbulent flow in a rectangular channel), the k_f can be calculated via

$$\frac{k_\mathrm{f} d_\mathrm{h}}{D_\infty} = 0.04\mathrm{Re}^{0.75}\mathrm{Sc}^{0.33} \quad (8)$$

where Re is the Reynolds number; Sc is the Schmidt number; d_h is the hydraulic diameter of the channel.

Thus, by fitting the achieved R_r with J_V, λ and L/ϵ can be determined for each combination of membrane and solute. In reality, the fitting was conducted at two sequential steps. First, the model parameter ΦK_c and Pe/J_V which are independent of J_V were calculated by fitting the retention data (R_r with J_V) to Eq. 2 using an optimization procedure (Solver, Microsoft Excel). Then the ΦK_c achieved which is only a function of λ was used to calculate the membrane pore radius.

Supplementary Note 3.

The salt permeation rate through PA TFC membranes was measured with two typical RO membranes (SW30 and XLE with respective pore diameters of 5.7 and 5.9 Å; Supplementary Note 2, Supplementary Figs. 9A and 9B), a tight nanofiltration (NF) membrane (NF90 with a pore diameter of 6.8 Å; Supplementary Fig. 9C), a loose NF membrane (NF270 with the pore diameter of 7.5 Å; Supplementary Fig. 9D), and two tight UF membranes (UA60 and NDX with respective pore diameters of 9.7 and 10.4 Å; Supplementary Figs. 9E and 9F). The NaCl/Na_2SO_4 and NaCl/$CaCl_2$ selectivities for these membranes, obtained from salt permeation experiments using the diffusion cell, are summarized in Fig. 3D.

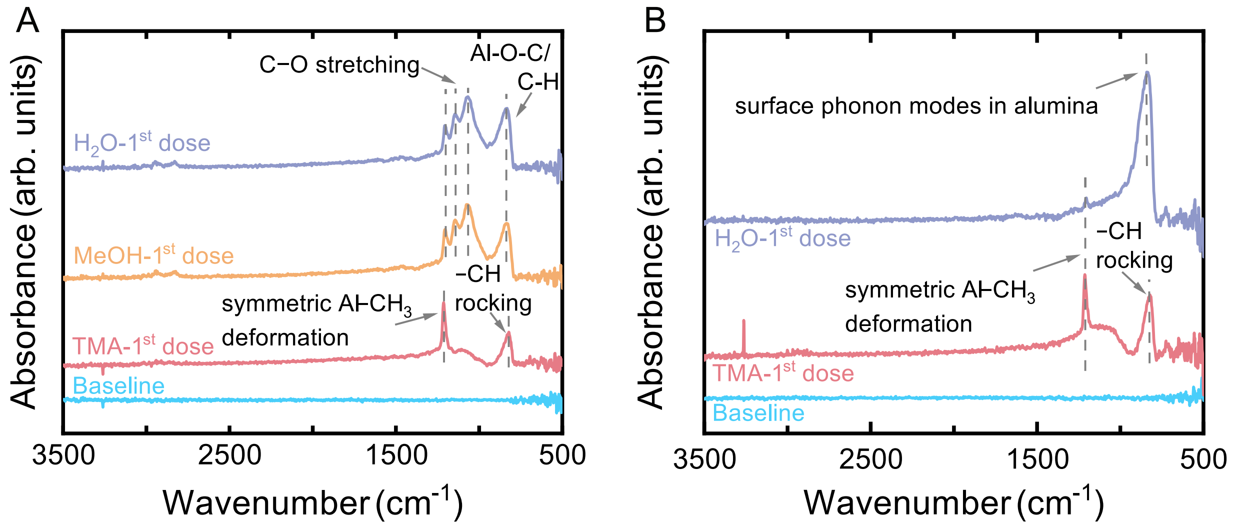

Supplementary Figure 1. (A) Original FTIR spectra recorded during the CH_3OH-modulated MeO-AlO_x ALD deposition after one pulse of TMA (red), methanol (orange), and H_2O (purple). (B) Original FTIR spectra recorded during the conventional Al_2O_3 ALD deposition after one pulse of TMA (red) and H_2O (purple).

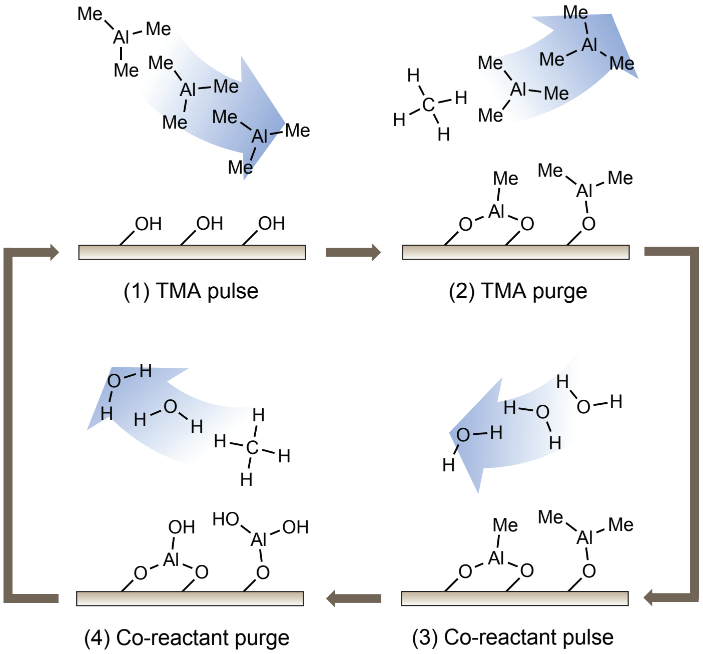

Supplementary Figure 2. Schematic diagram illustrating the conventional Al_2O_3 ALD deposition, which consists of four steps: (A) TMA pulse; (B) TMA purge; (C) Co-reactant pulse (i.e., H_2O); and (D) Co-reactant purge. Only a single atomic layer of Al_2O_3 is deposited during one ALD cycle.

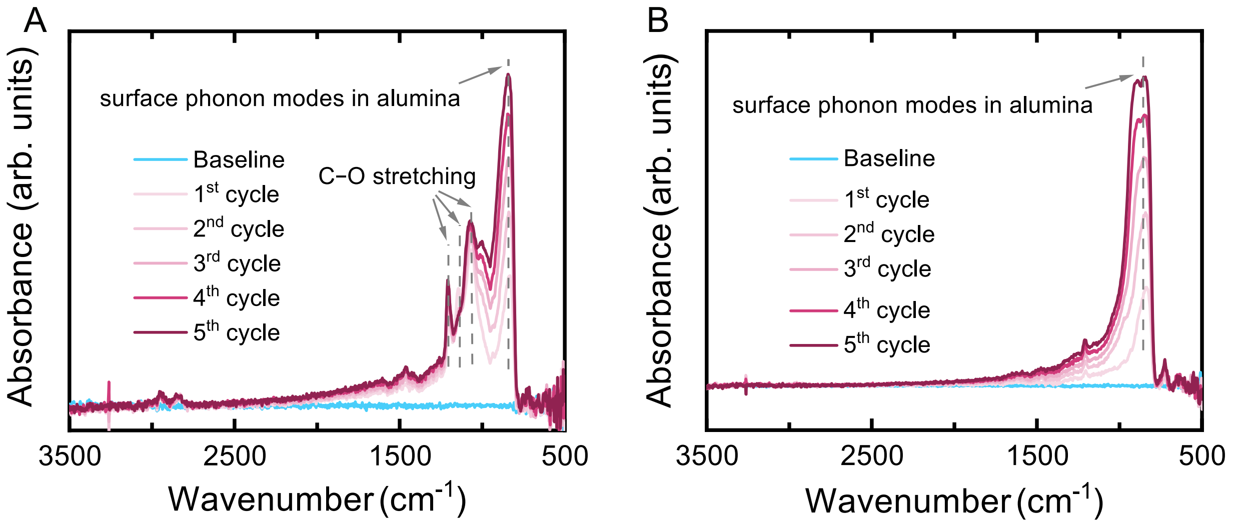

Supplementary Figure 3. (A) FTIR spectra recorded during the first five cycles of (A) the CH_3OH-modulated MeO-AlO_x ALD deposition and (B) the conventional Al_2O_3 ALD deposition.

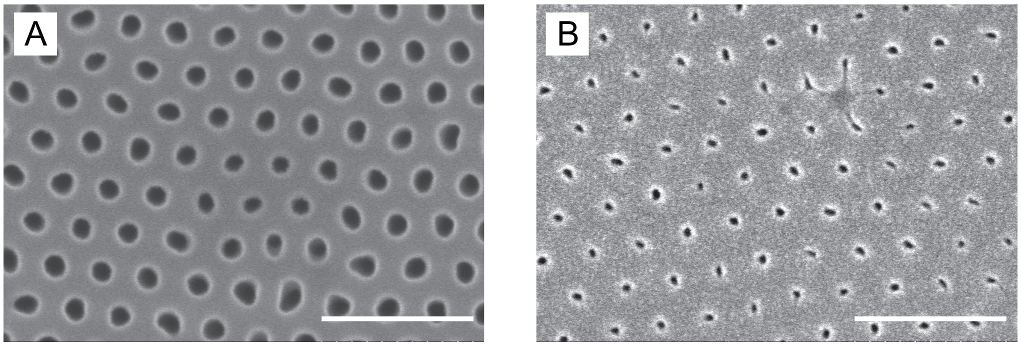

Supplementary Figure 4. SEM images of the (A) pristine AAO substrates and (B) AAO substrates undergoing 120 cycles of the conventional Al_2O_3 ALD modification. Scale bars are 200 nm.

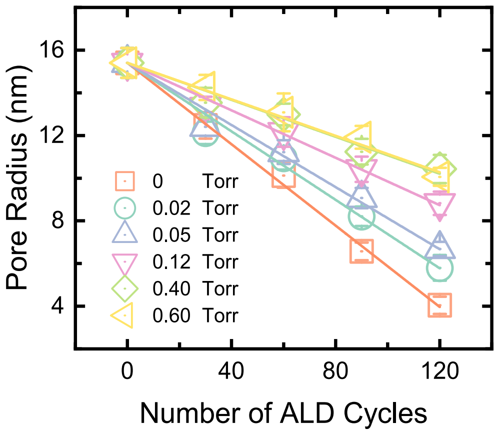

Supplementary Figure 5. Dependence of the pore radius of the AAO substrates on the number of ALD cycles conducted. In each ALD deposition, the TMA and H_2O dosages were fixed at 0.25 and 0.06 Torr, respectively. The CH_3OH dosage was increased from 0 to 0.02, 0.05, 0.12, 0.40, and 0.60 Torr. Error bars represent standard deviations from measurements with at least thirty pores.

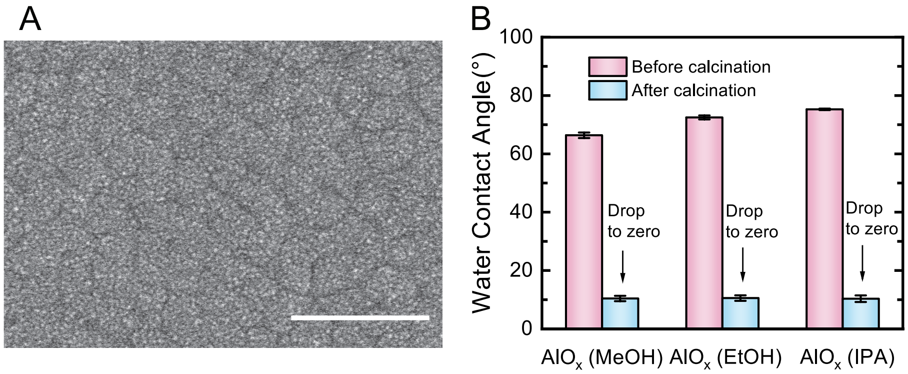

Supplementary Figure 6. (A) SEM images of the MeO-AlO_x ALD film on top of the AAO substrates. Scale bars are 200 nm. (B) The water contact angle of the AlO_x membranes before (pink) and after (blue) calcination. Error bars represent standard deviations of at least 3 measurements.

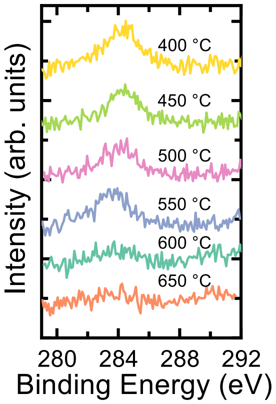

Supplementary Figure 7. C 1*s* XPS spectra of the MeO-AlO_x ALD film after being calcinated at 400, 450, 500, 550, 600, and 650 °C.

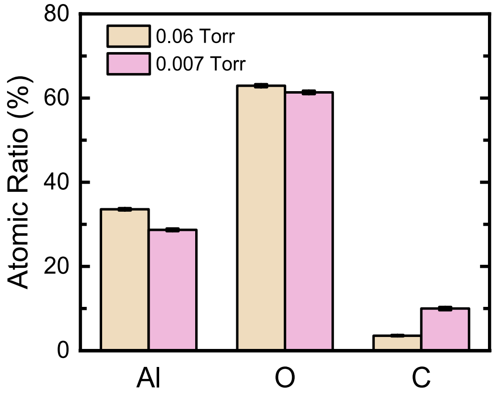

Supplementary Figure 8. Atomic ratio of Al, O, and C within the MeO-AlO_x ALD films deposited with 0.06 (beige) and 0.007 (pink) Torr H_2O dosage. Error bars represent the standard deviations of 5 measurements.

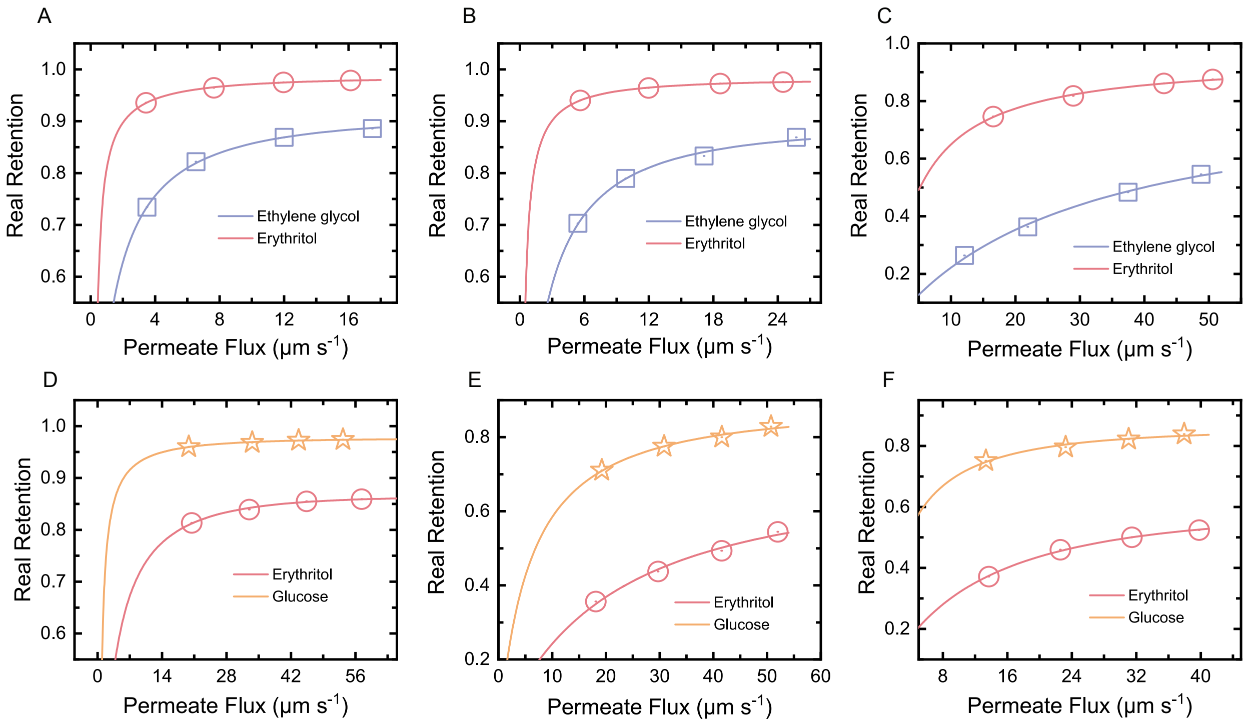

Supplementary Figure 9. Dependence of membrane real retention of neutral organic tracers on membrane permeate water flux. Ethylene glycol and erythritol were utilized for the measurements with (A) SW30, (B) XLE, and (C) NF90 membranes, and erythritol and glucose were chosen for (D) NF270, (E) NDX, and (F) UA60 membranes. Experiments were conducted using a bench-scale crossflow filtration system. In each experiment, a single organic tracer was used at a feed concentration of 50 mg/L (as TOC). Permeate samples were collected under pressures of 6.9, 13.8, 20.7, and 27.6 bar for SW30, XLE, and NF90 membranes and of 6.9, 10.3, 13.8, and 17.2 bar for NF270, NDX, and UA60 membranes. Fitting is based on the membrane hindrance transport model. In all the experiments, temperature was maintained at 22.0 ± 0.5 °C and the crossflow velocity was set at 21.4 cm/s.

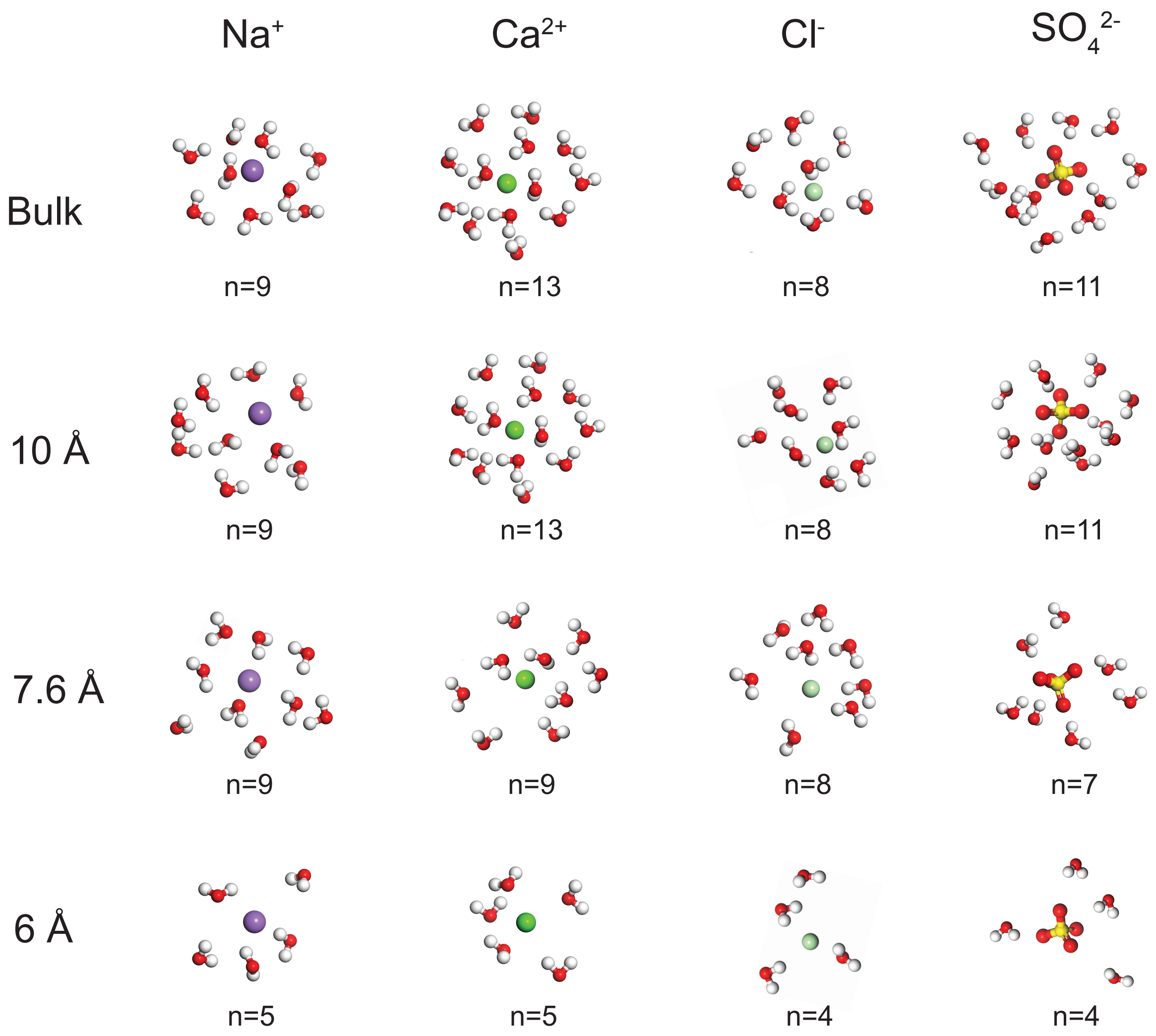

Supplementary Figure 10. Hydration state of representative Na^+, Ca^{2+}, Cl^-, and SO_4^{2-} ions in the bulk solution and inside the pores with diameters of 10, 7.6, and 6 Å.

Supplementary References

1. Elam JW, Routkevitch D, Mardilovich PP, George SM. Conformal coating on ultrahigh-aspect-ratio nanopores of anodic alumina by atomic layer deposition. *Chemistry of Materials* **15**, 3507-3517 (2003).
2. Nghiem LD, Schafer AI, Elimelech M. Removal of natural hormones by nanofiltration membranes: Measurement, modeling, and mechanisms. *Environmental Science & Technology* **38**, 1888-1896 (2004).
3. Xie M, Nghiem LD, Price WE, Elimelech M. Relating rejection of trace organic contaminants to membrane properties in forward osmosis: Measurements, modelling and implications. *Water Research* **49**, 265-274 (2014).
4. Bungay PM, Brenner H. The motion of a closely-fitting sphere in a fluid-filled tube. *International Journal of Multiphase Flow* **1**, 25-56 (1973).
